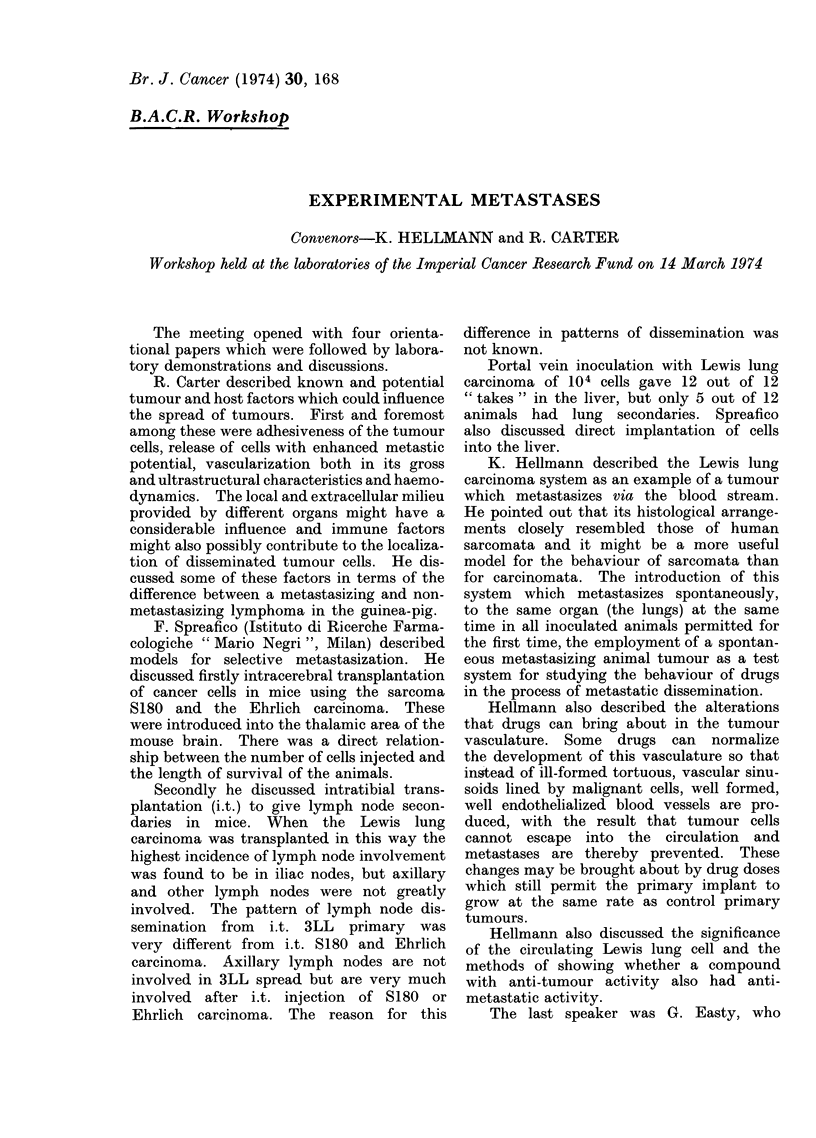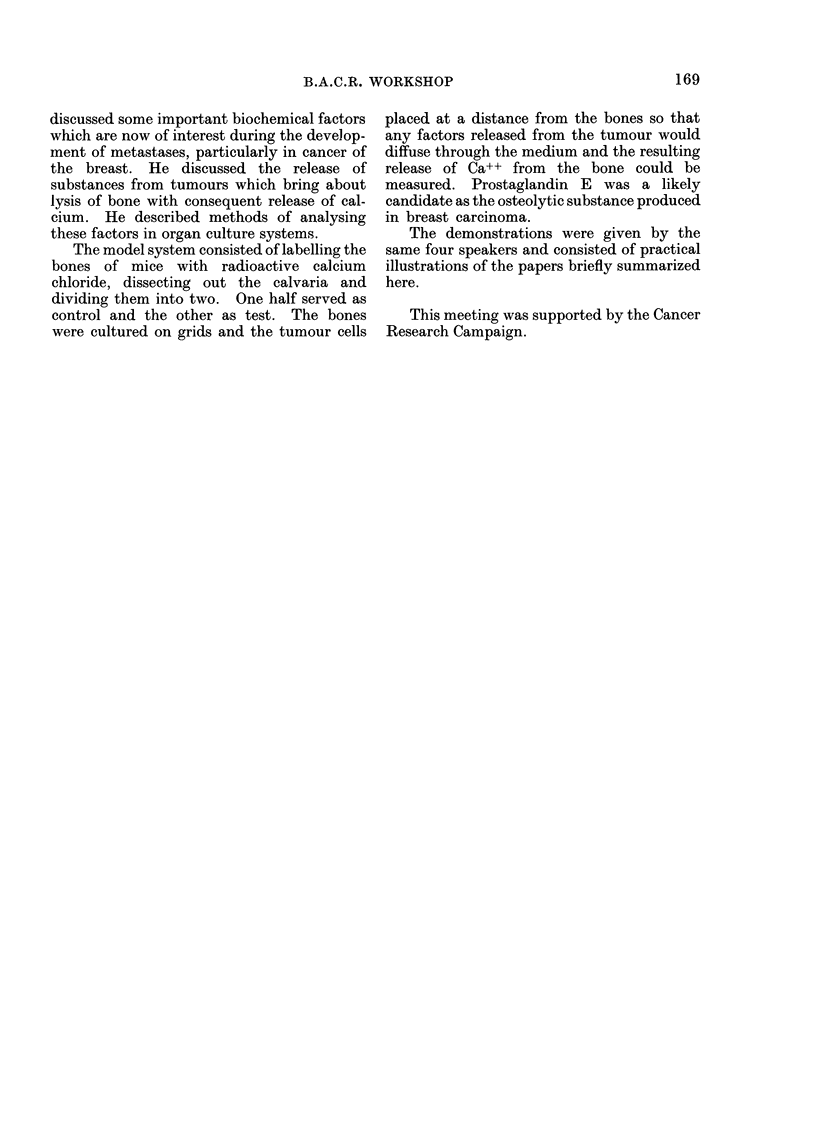# Experimental Metastases

**Published:** 1974-08

**Authors:** 


*Br. J. Cancer* (1974) **30**, 168

***B.A.C.R. Workshop***

### EXPERIMENTAL METASTASES

*Convenors*—K. HELLMANN and R. CARTER

*Workshop held at the laboratories of the Imperial Cancer Research Fund on 14 March 1974*

The meeting opened with four orientational papers which were followed by laboratory demonstrations and discussions.

R. Carter described known and potential tumour and host factors which could influence the spread of tumours. First and foremost among these were adhesiveness of the tumour cells, release of cells with enhanced metastic potential, vascularization both in its gross and ultrastructural characteristics and haemodynamics. The local and extracellular milieu provided by different organs might have a considerable influence and immune factors might also possibly contribute to the localization of disseminated tumour cells. He discussed some of these factors in terms of the difference between a metastasizing and nonmetastasizing lymphoma in the guinea-pig.

F. Spreafico (Istituto di Ricerche Farmacologiche " Mario Negri ", Milan) described models for selective metastasization. He discussed firstly intracerebral transplantation of cancer cells in mice using the sarcoma S180 and the Ehrlich carcinoma. These were introduced into the thalamic area of the mouse brain. There was a direct relationship between the number of cells injected and the length of survival of the animals.

Secondly he discussed intratibial transplantation (i.t.) to give lymph node secondaries in mice. When the Lewis lung carcinoma was transplanted in this way the highest incidence of lymph node involvement was found to be in iliac nodes, but axillary and other lymph nodes were not greatly involved. The pattern of lymph node dissemination from i.t. 3LL primary was very different from i.t. S180 and Ehrlich carcinoma. Axillary lymph nodes are not involved in 3LL spread but are very much involved after i.t. injection of S180 or Ehrlich carcinoma. The reason for this difference in patterns of dissemination was not known.

Portal vein inoculation with Lewis lung carcinoma of $10^4$ cells gave 12 out of 12 " takes " in the liver, but only 5 out of 12 animals had lung secondaries. Spreafico also discussed direct implantation of cells into the liver.

K. Hellmann described the Lewis lung carcinoma system as an example of a tumour which metastasizes *via* the blood stream. He pointed out that its histological arrangements closely resembled those of human sarcomata and it might be a more useful model for the behaviour of sarcomata than for carcinomata. The introduction of this system which metastasizes spontaneously, to the same organ (the lungs) at the same time in all inoculated animals permitted for the first time, the employment of a spontaneous metastasizing animal tumour as a test system for studying the behaviour of drugs in the process of metastatic dissemination.

Hellmann also described the alterations that drugs can bring about in the tumour vasculature. Some drugs can normalize the development of this vasculature so that instead of ill-formed tortuous, vascular sinusoids lined by malignant cells, well formed, well endothelialized blood vessels are produced, with the result that tumour cells cannot escape into the circulation and metastases are thereby prevented. These changes may be brought about by drug doses which still permit the primary implant to grow at the same rate as control primary tumours.

Hellmann also discussed the significance of the circulating Lewis lung cell and the methods of showing whether a compound with anti-tumour activity also had antimetastatic activity.

The last speaker was G. Easty, who discussed some important biochemical factors which are now of interest during the development of metastases, particularly in cancer of the breast. He discussed the release of substances from tumours which bring about lysis of bone with consequent release of calcium. He described methods of analysing these factors in organ culture systems.

The model system consisted of labelling the bones of mice with radioactive calcium chloride, dissecting out the calvaria and dividing them into two. One half served as control and the other as test. The bones were cultured on grids and the tumour cells placed at a distance from the bones so that any factors released from the tumour would diffuse through the medium and the resulting release of $Ca^{++}$ from the bone could be measured. Prostaglandin E was a likely candidate as the osteolytic substance produced in breast carcinoma.

The demonstrations were given by the same four speakers and consisted of practical illustrations of the papers briefly summarized here.

This meeting was supported by the Cancer Research Campaign.